# Development and Validation of a Western Blot Method to Quantify Mini-Dystrophin in Human Skeletal Muscle Biopsies

**DOI:** 10.1208/s12248-022-00776-0

**Published:** 2022-12-20

**Authors:** Catherine I. Soderstrom, Jennifer Larsen, Carolina Owen, David Gifondorwa, David Beidler, Florence H. Yong, Patricia Conrad, Hendrik Neubert, Steven A. Moore, Mohamed Hassanein

**Affiliations:** 1 Early Clinical Development, Precision Medicine, Cambridge, MA, USA; 2 Clinical Assay Group, Global Product Development (GPD), Pfizer Inc, Groton, Connecticut, USA; 3 Early Clinical Development, Precision Medicine, Pfizer Inc., 1 Portland, Cambridge, Massachusetts 02139, USA; 4 Biostatistics, Early Clinical Development, Worldwide Research & Development, Pfizer Inc., Cambridge, MA, USA; 5 Biomedicine Design, Worldwide Research & Development, Pfizer Inc., Andover, Massachusetts, USA; 6 Senator Paul D. Wellstone Muscular Dystrophy Specialized Research Center, Department of Pathology, Roy J. and Lucille A. Carver College of Medicine, The University of Iowa, Iowa City, Iowa 52242, USA

**Keywords:** Western blot, Quantitative, AAV9, Gene therapy, Duchenne muscular dystrophy

## Abstract

Duchenne muscular dystrophy (DMD) is a degenerative muscular disease affecting roughly one in 5000 males at birth. The disease is often caused by inherited X-linked recessive pathogenic variants in the *dystrophin* gene, but may also arise from de novo mutations. Disease-causing variants include nonsense, out of frame deletions or duplications that result in loss of dystrophin protein expression. There is currently no cure for DMD and the few treatment options available aim at slowing muscle degradation. New advances in gene therapy and understanding of dystrophin (DYS) expression in other muscular dystrophies have opened new opportunities for treatment. Therefore, reliable methods are needed to monitor dystrophin expression and assess the efficacy of new therapies for muscular dystrophies such as DMD and Becker muscular dystrophy (BMD). Here, we describe the validation of a novel Western blot (WB) method for the quantitation of mini-dystrophin protein in human skeletal muscle tissues that is easy to adopt in most laboratory settings. This WB method was assessed through precision, accuracy, selectivity, dilution linearity, stability, and repeatability. Based on mini-DYS standard performance, the assay has a dynamic range of 0.5–15 ng protein (per 5 μg total protein per lane), precision of 3.3 to 25.5%, and accuracy of − 7.5 to 3.3%. Our stability assessment showed that the protein is stable after 4 F/T cycles, up to 2 h at RT and after 7 months at − 70°C. Furthermore, our WB method was compared to the results from our recently published LC–MS method.

## Introduction

Muscular dystrophies are inherited disorders that are primarily characterized by progressive muscular weakness and wasting. The different types of dystrophies present a rather heterogenous severity disease progression and are subcategorized into several groups based on disease etiology (1–3). Most often the different types of muscular dystrophies are characterized by the muscles they affect which has led to a better understanding of the underlying genetics involved in the disease progression (4).

Of the many known muscular dystrophies, two types involve pathogenic variants in the X-linked *dystrophin* (*DMD*) gene (5, 6). Duchenne muscular dystrophy (DMD) is the most common. DMD patients exhibit little to no expression of dystrophin (DYS), due to pathogenic *DMD* variants that result in loss of dystrophin expression (7). Becker muscular dystrophy (BMD) is less common and less severe than DMD (8, 9). BMD is allelic to DMD, resulting from milder pathogenic variants in *DMD*. Most BMD-associated variants are in frame deletions or duplications. The resulting protein is shortened (or lengthened), but partially functional. Abnormalities in dystrophin protein expression leave both BMD and DMD patients with reduced muscle fiber integrity, leading to life-long, repetitive cycles of myonecrosis and regeneration, endomysial fibrosis (scarring), and eventual fatty replacement of muscle (5, 10).

Until recently, many muscular dystrophies have been considered to be untreatable genetic disorders. However, since 1974, several treatment modalities have been identified and used, including prednisone and other glucocorticoids (11–13). The emergence of a recombinant adeno-associated virus (AAV) that has the potential of providing long-lasting expression of a designed, short version of the dystrophin protein offered new hope for restoring functional dystrophin expression. Gene transfer therapy using AAV vectors has shown to be particularly efficient in transducing skeletal muscle fibers and cardiomyocytes when packaged with the appropriate capsid and allow long-term in vivo transgene expression in mouse and dog pre-clinical models (14–18). Based on these initial proof of concept results from pre-clinical models, there are currently forty ongoing clinical trials on DMD using gene therapy approach as listed in clinicaltrials.gov.

Historically, immunohistochemistry and Western blotting (WB) have been common methods used for dystrophin protein analysis (19, 20). However, these methods were more suitable for exploratory basic science research rather than clinical trial sample testing (21, 22). Traditional WB methods that are qualitative or semi-quantitative suffered from several limitations that hindered utility of WB as an accurate and quantitative bioanalytical tool for measuring biomarkers in support of clinical trial studies. These factors include lack of reproducibility, inaccuracies due to assay variability, inappropriate normalization methods, and ill-considered selection of stably expressed housekeeping proteins (HKPs) (23–28). With the recent number of AAV gene therapy trials for DMD, there is a bioanalytical need for reliable, robust, and reproducible assays to quantify dystrophin (29, 30).

Here, we describe the validation of a fit-for-purpose WB method for quantitative measurement of AAV-transduced or recombinant mini-dystrophin (mini-DYS) in human muscle tissues that is simple, affordable, reproducible and can be easily performed in regular laboratory settings (31). Mini-DYS is the truncated, functional, and therapeutic form of the full-length dystrophin protein. As such, mini-DYS is an accepted functional surrogate for full-length DYS protein (32). Our WB method utilizes a standard curve of recombinant mini-dystrophin protein as a reference standard to measure both endogenous DYS proteins (both full-length and certain truncated proteins) and mini-DYS expressed by AAV9. Therefore, this assay is a quantitative assay for measuring the AAV9-transduced mini-DYS in muscle tissue lysates while it can be used as quasi-quantitative for endogenous full-length DYS protein. We also show that the methods can be used for quasi-quantitation of full-length wild-type (wt) dystrophin. Our assay was developed and validated to overcome some of the limitations of previous WB methods with potential use to support clinical trial studies in mind. This started with the careful selection of glyceraldehyde-3-phosphate dehydrogenase (GAPDH) to be the most appropriate HKP as loading control. We show that it is stably expressed in DMD samples from animal models and humans (17, 33, 34). In addition, our validation plan incorporated quality controls and calibration standard of human recombinant mini-dystrophin protein spiked in a pool of normal skeletal muscle lysates and evaluated the assay parameters for inclusion in validation testing. We evaluated the assay performance in terms of precision and accuracy using spike quality control (QC) samples, dilutional linearity, reproducibility (multiple analysts, different days), and matrix effect. This method provides an affordable and reliable platform for analyzing clinical samples from AAV gene therapy studies in a typical research laboratory setting. Data generated using this method were compared to the results from orthogonal analysis of the same samples (20 healthy, 20 BMD, and 20 DMD) using a liquid chromatography–mass spectrometry (LC–MS) method (32).

## Materials and Methods

### Assay Development

Methods were provided by Dr. Francesco Muntoni (King’s College, London), and methods transferred from Bamboo Therapeutics Inc., Chapel Hill, NC. Selection of lysis buffer, control, and anti-dystrophin antibodies was largely based upon their earlier development work (35). Lysis methods, total protein determination, gel loading, and transfer steps were optimized during development and validation (35).

### Sample Preparation

Skeletal muscle lysates were prepared in DMD lysis buffer containing 4 M Urea, 125 mM Tris, 4% SDS, 10% glycerol, and 10% ß-mercaptoethanol + Roche cOmplete-EDTA-free^™^ protease inhibitor cocktail tablet (1 tablet per 10 mL buffer, Roche Diagnostics GmbH, Mannheim, Germany). Ten-micron sections of skeletal muscle were homogenized with 150 μL of DMD lysis buffer on ice in microcentrifuge tubes using a pellet pestle motor homogenizer. After homogenization, samples were rotated in a MACSmixtube rotator at 4°C for 2 h. Samples were vortexed and then spun at 20,000 rpm in a microcentrifuge at 4°C for 20 min to pellet insoluble material. Supernatants were aliquoted and stored frozen at − 70°C until use.

### Total Protein Analysis

Total protein standards were prepared by diluting 5 mg/mL bovine serum albumin (BSA) in DMD lysis buffer, and then diluting serially 1:2 six times (5–0.078 mg/mL). Test samples were thawed on ice. Aliquots were removed for total protein measurement. A total of 1 μL of each test sample or BSA standard was pipetted onto a nitrocellulose membrane, at least in duplicate measurement. After air drying, the membrane was incubated with 1X LI-COR REVERT^™^ stain (LI-COR, Lincoln, NE) as per the vendor protocol. The membrane was incubated in the stain for 5 min, and then washed twice in equal volumes of 1X LI-COR REVERT^™^ Wash Buffer. The dots on the membrane were visualized and quantitated on a LI-COR Odyssey CLX^™^ Imaging System (LI-COR, Lincoln, NE). Total protein on the samples was determined by interpolation of dot-blot fluorescence intensities into the BSA standard curve as described previously (36, 37).

### Recombinant Mini-Dystrophin

Recombinant mini-dystrophin protein was produced by transfection of a flag-tag version of the PF-06939926 (fordadistrogene movaparvovec) capsid into HEK293 cells (38), and growth of the cells in culture, followed by lysis of cells followed by affinity purification (Kemp Biologics, Frederick, MD).

### Calibration and Quality Control Sample Preparation

All calibration standards and quality controls (QC) were prepared by spiking purified recombinant mini-dystrophin (Kemp Biologics, Frederick, MD) into a pooled healthy skeletal muscle lysate with total protein ranging from 0.1 to 2.0 mg/mL, prepared from bicep muscle of four healthy adult donors (obtained from Pfizer’s Tissue Bank) (14, 39). Calibration standards and quality controls were freshly prepared using mini-dystrophin (mini-DYS) from two separate, independent dilutions of recombinant protein. Standards were prepared at 0, 0.102, 0.256, 0.64, 1.60, 4.0, 10.0, and 15.0 ng of mini-dystrophin per 5 μg of muscle lysate protein (*n* = 1, *i.e*., one lane each per gel). The same preparation method was utilized for QCs by spiking mini-DYS into skeletal muscle lysate pool at 0.5 ng (LLOQ1), 1.0 ng (LLOQ2), 2.5 ng (LQC), 5.0 ng (MQC), 8.0 ng (HQC), and 12.0 ng (ULOQ) of mini-DYS per 5 μg of muscle lysate protein.

### Gel Electrophoresis and Western Blotting

Each calibration standard, QC, or test sample was run on a 3–8% Tris–Acetate SDS-PAGE with 1X Tris–Acetate SDS Running Buffer (Novex, Waltham, MA); 5 μg total protein per lane was loaded. The gels were transferred to nitrocellulose membranes using a tank transfer apparatus. The membranes were probed for mini-dystrophin protein using the Leica Biosystems^™^ (Buffalo Grove, IL) detection antibody DYSB (exons 10–12) diluted at 1:150 and rabbit anti-GAPDH at 1:10,000 Abcam (Branford, CT) and visualized with LI-COR Odyssey fluorescent labeled anti-mouse and anti-rabbit antibodies at 1:15,000 at 700 nm (DYS and mini-DYS) and 800 nm (GAPDH) channels.

### Human Skeletal Muscle Samples

Muscle samples for the study came predominantly from a tissue repository at the Iowa Senator Paul D. Wellstone Muscular Dystrophy Specialized Research Center (MDSRC). Residual frozen tissue from diagnostic muscle biopsies was available for research as approved by the local institutional research ethics review board of the University Iowa, IRB protocol #200,510,769, title “Muscle Biopsy/Cell Repository/Diagnostics Core,” original approval date 02/16/2006, latest continuing review approval date 07/11/2022. Biopsies with a pathology report diagnosis of “no diagnostic abnormality” were used as normal controls (*n* = 20). Biopsies evaluated by immunofluorescence and determined to be dystrophinopathy cases were assigned to Becker muscular dystrophy (BMD, *n* = 20) or Duchenne muscular dystrophy (DMD, *n* = 20) based on clinical presentation, age at the time of muscle biopsy, severity of dystrophic pathology, dystrophin expression, and, when available, the *DMD* molecular genetic testing results. Eight of 20 control biopsies were from females; all the remaining biopsies were from males. The age ranges were 4 to 18 years for controls, 3 to 19 years for BMD, and 3 to 10 years for DMD. Nineteen of 20 control biopsies were from the quadriceps muscle; the remaining biopsy was from gastrocnemius. All 20 BMD biopsies and 18 of 20 DMD biopsies were from the quadriceps; the sites for the remaining 2 DMD biopsies were not provided with the pathology requisitions. This muscle sample set was defined as “20/20/20” (*n* = 20 for each cohort of normal controls, BMD, and DMD). Additional control human skeletal muscle samples were obtained from ten (10) healthy subjects from the Pfizer Tissue Bank repository to prepare a tissue lysate pool for the constitution of standard curve and QCs.

### Data Acquisition and Signal Quantitation

The raw light counts of mini-dystrophin were determined using a LI-COR Odyssey CLX^™^ Imager and the image analysis was performed using Image Studio^®^ Software version 5.3, 2015. Raw signal file was imputed into biomarker analysis tool Watson LIMS(R). Dystrophin and mini-dystrophin signals were quantitated in the green fluorescent channel (800 nm) and GAPDH was quantitated in the red fluorescent (700 nm) channel, respectively. Relative fluorescence units (RFU) were generated by drawing boxes around each appropriate band keeping the size/integration area as constant as possible. The fluorescent signal from each mini-dystrophin band was normalized (divided) by the housekeeping glyceraldehyde-3-phosphate dehydrogenase (GAPDH) signal as a single-protein loading control. The normalized values were then analyzed against nominal concentrations of mini-DYS standards in Watson LIMS 7.5 (Thermo Scientific), using logistic (auto estimate) regression and 1/*y* weighting factor.

### Statistical Analysis

For all assay validation data tables, final concentrations were reported in nanograms (ng) of protein, to three significant figures. Precision, defined by the coefficient of variation (%CV), and accuracy, defined by relative error (%RE), were reported to the nearest 0.1%. Rounding was performed on all values exported from Watson version 7.5 and the subsequent calculations using those values. Statistical results were reported in a manner consistent with Watson LIMS systems. The following terminology is considered equivalent: %CV = Relative Standard Deviation, RSD = Precision (%), %RE = %, %Bias = Accuracy (%). The assay is considered acceptable if the overall precision (%CV) and accuracy (%RE) ≤30.0% for all STD curve and QC except for LLOQ and ULOQ where ≤50% was acceptable.

Relationship between the dystrophin levels obtained by WB and previously published LC–MS results (32) was evaluated using the “20/20/20” muscle samples collected from 20 normal controls and 20 BMD and 20 DMD patients (40–42). The WB method was validated for the quantitation of mini-DYS in diseased patient samples. The WB method and the use of mini-DYS standard were used to measure the levels of dystrophin as surrogate protein in the DMD, BMD, and healthy tissue samples. Regression analysis was conducted to examine the association between the results obtained by WB and LC–MS methods. The adjusted *R*^2^, a measure of the goodness-of-fit for the regression models identifying the percentage of variance in the dystrophin levels obtained by LC–MS explained by a set of input variable(s), will be reported. Spearman’s rank correlation was used to assess monotonic relationships and Pearson’s correlation was used to assess linear relationships. Bootstrap methods were used to obtain the 95% confidence intervals for the mean based on 10,000 bootstrap samples of available data in each cohort. Dystrophin concentrations below the LLOQ of 0.1 ng/μg protein (BLQ) obtained by WB were imputed as 0.5*LLOQ, based on the uniform distribution in the range of 0 to 0.1 ng/μg. Corresponding values relative to the average of the control group were computed using the imputed values (0.05 ng/μg) relative to the average dystrophin levels of the respective pool by WB. The imputed BLQ values were included in the summary statistics and graphical presentation unless otherwise specified.

## Results

### Assay Validation

**Fig. 1:**
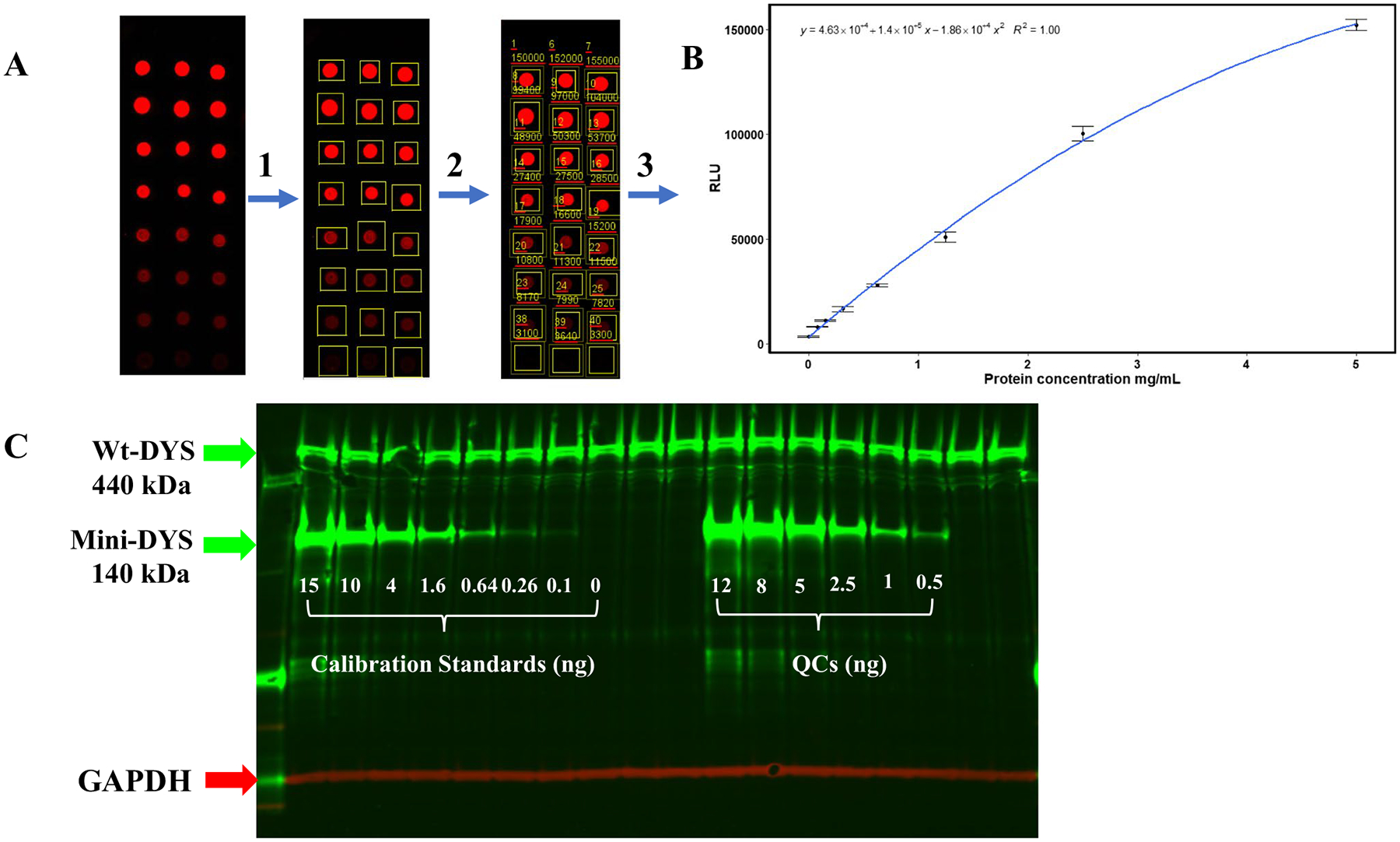
Quantitation of dystrophin using mini-dystrophin STD curve. A representative scheme of a validation run. **A** Image acquisition and quantitation of total protein from tissue extracts using dot-blot followed by **B** construction of standard curve. **C** An image of a WB gel showing the wt-DYS, mini-DYS standard curve, quality controls (QCs) detected through the green fluorescent channel (800 nm), and protein normalization with GAPDH using the 700 nm channel (in red) is shown

Here, we report the validation of a WB method for accurate quantitation of mini-dystrophin protein in human skeletal muscle tissue lysates. The assay utilizes a calibration standard (STD) that was prepared by spiking purified recombinant mini-dystrophin (mini-DYS) in a pool of skeletal muscle lysates collected from normal individuals. Fluorescence signals acquired from a LI-COR WB imager for all tissue lysate samples and quality controls (QC) were first normalized to a housekeeping protein, GAPDH, and then plotted against the calibration standard to interpolate the DYS concentrations (see representative of an analytical run in Fig. 1A and B.

#### STD

Our WB method was validated with the potential for clinical use in mind. As such, we set up a rigorous assay performance evaluation plan that included the following parameters: (1) precision and accuracy (intra- and inter-run); (2) sensitivity/dynamic range; (3) matrix testing; (4) dilution linearity; and (5) stability, including bench top, freeze–thaw, and long-term freezer stability (Supplemental Table II).

##### Precision and Accuracy

In total, twenty WB runs were performed over 119 days during the course of the assay validation. All acceptable runs were included in the precision and accuracy assessments (Tables I and II; Supplemental Tables I and II). A maximum of two precision and accuracy runs per day included freshly prepared mini-DYS standard curves and at least one replicate of quality controls at HQC, MQC, and LQC. In each primary validation batch, the following mini-DYS samples were processed at 0.5 ng (LLOQ1), 1.0 ng (LLOQ2), 2.5 ng (LQC), 5.0 ng (MQC), 8.0 ng (HQC), and 12.0 ng (ULOQ) spiked into a pool of human muscle lysate.

Two LLOQ levels were assessed to evaluate assay sensitivity, 0.5 ng and 1.0 ng mini-DYS. Per assay validation’s criteria, only LLOQ2 was acceptable. Values under 1.0 ng mini-DYS were not reported. Accuracy was assessed by the difference (%) between calculated concentration of each standard and QC and their respective nominal concentration (see the “Materials and Methods” section). Performance of back-calculated standards is shown in Table I. The overall assay precision (%CV) of QCs ranged from 13.3 to 22.8% with an accuracy of − 4.2 to 42% at LLOQ1 (0.5) and 8% at LLOQ2 (1.0 ng included) based on standard curve performance (see a representative of the standard curve in Supplemental Fig. 1).

The assay precision was assessed through the performance of %CV defined as follows: (Mean Conc / Std-Dev) × 100. Samples were acceptable when %CV was ≤ 30%. Accuracy was assessed through the performance of %RE (relative error) defined as the difference (%) between the back-calculated concentration and nominal concentration of the STD or QC sample. The validation package acceptance criteria as stated in the method validation plan for linearity, accuracy, precision, and stability were met as shown in Tables I and II.

##### Dynamic Range

The mini-DYS standard curve showed a linear dynamic range of 0.102–15.0 ng of recombinant mini-DYS spiked into 5 μg total protein per lane (0.0204–3 ng/μg protein final), with two LLOQs (Lower Limit of Quantitation) tested to challenge the assay’s LLOQ at 0.5 ng (equivalent to 0.1 ng/μg of protein) and 1.0 ng of loaded protein, and an ULOQ (Upper Limit of Quantitation) of 12.0 ng (2.4 ng/μg). The other QCs were set at 1.0 ng (LQC, 0.2 ng/μg), 2.5 (LQC2, 0.50 ng/μg), 5.0 (MQC, 1.0 ng/μg), and 8.0 (HQC, 1.6 ng/μg) (Tables I and II; Supplemental Table I).

##### Dilution Linearity

Briefly, human skeletal lysates from three individuals (*n* > 3) were spiked with purified recombinant mini-DYS at 1, 16, and 50 ng, and further diluted at 2-, 4-, 10-, and 20-fold in DMD lysis buffer to assess assay linearity of dilution and matrix effects. The results depicted in Table III indicated that two (2) out of three (3) spike preparations showed linearity until 20-fold dilution, and 3 out of 3 preparations were linear at fourfold dilution. These results demonstrate that sample-dilution response curve of recombinant mini-DYS has similar immunoreactivity to the standard-calibrator response curve in the presence of endogenous protein. As such, this further confirms that mini-DYS is suitable for use as a calibrator on this assay.

##### Stability of Mini-DYS

Assessing analyte stability in its native matrix is critical for the successful implementation of bioanalytical assays in clinical settings. In addition, there has been more scrutiny in recent years from the regulatory authorities on the proper examination of stability for bioanalytical assays intended for clinical use (43). We therefore set to evaluate analyte stability in a manner that mimics real-life sample handling and storage conditions. First is the freeze/thaw (F/T) stability of recombinant mini-dystrophin protein using spiked human skeletal muscle lysates at LQC (2.5 ng mini-DYS) and HQC (8.0 ng mini-DYS). Each sample was subjected to at least 4 F/T cycles at − 70°C, with a duration of at least 24 h on the first frozen cycle (− 70°C). For, subsequent F/T cycles, samples were frozen for a minimum of 12 h at − 70°C with a thawing duration of at least 45 min at room temperature (RT). Samples were analyzed together after the last F/T cycle against freshly prepared spiked controls. The stability tests on recombinant mini-DYS spiked from three pools of healthy skeletal muscle at low and high QC levels showed that samples were stable for up to 2 h at room temperature. Long-term stability was acceptable up to 7 months of frozen storage (− 70°C) at LQC and HQC while F/T stability was demonstrated for up to 4 cycles (Table IV).

### Human Sample Analysis

**Fig. 2:**
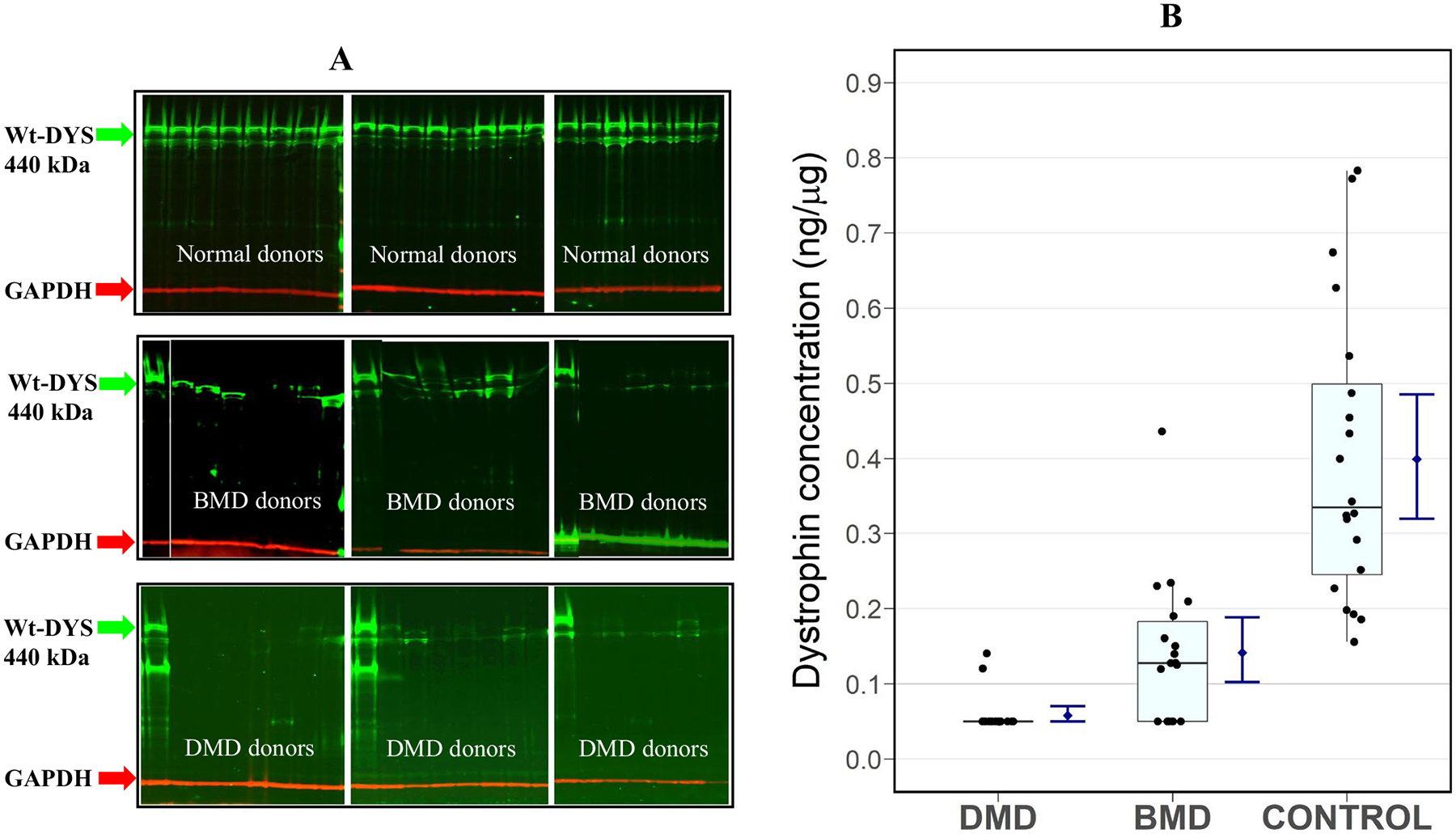
Quantitation of dystrophin in healthy and diseased human muscle tissues. **A** Gel electrophoresis images of wt-DYS from 20 normal, 18 BMD, and 20 DMD donors. **B** Dystrophin concentration quantified by Western blot in 3 cohorts with 20 subjects each in DMD and non-dystrophic control, and 18 subjects in the BMD group. The lower and upper hinges of the boxplot correspond to the first and third quartiles (the 25th and 75th percentiles); the upper whisker extends from the hinge to the largest value no further than 1.5*IQR (inter-quartile range) from the hinge, and the converse is the case for the lower whiskers. Data beyond the end of the whiskers are outliers and are plotted individually. Blue bars are the 95% confidence intervals for the mean in each group (blue diamonds), based on 10,000 bootstrap samples of the sample size in each cohort (40, 54) (Table V)

Following the satisfactory completion of our WB method validation, 20 normal controls, 20 BMD, and 20 DMD patient muscle biopsy extracts were analyzed. Western blot analysis of these samples showed a broad range of wt dystrophin expression in normal healthy donors (Fig. 2A). Endogenous levels of dystrophin in human skeletal muscle tissue ranged from 0.16 to 0.78 ng/μg of total protein in the normal donors (*n* = 20) with an average of 0.40 ng/μg. Protein levels in samples of 12 BMD patients were 0.12–0.44 ng/μg; 6 samples were reported as BLQ, while two other samples did not have valid results. For the DMD cohort, 18 out of 20 samples were reported as BLQ, and two other DMD patients had protein levels of 0.12–0.14 ng/μg (Fig. 2B). The control pooled skeletal muscle for our validation methods was used as the 100% normal dystrophin and reported 0.44 ng dystrophin/μg total protein. The summary statistics of the dystrophin concentrations for each cohort including imputed values below the LLOQ of 0.1 ng/μg for 6 BMD and 18 DMD patients is presented in Table V.

The raw light counts of internal loading controls of mini-DYS spiked on healthy skeletal muscle lysates were analyzed using LI-COR Image Studio 5.3 and further normalized per GAPDH intensities on each lane. The normalization step was required to ensure accurate interpretation of the DYS concentration on each sample. One of the advantages of the LI-COR system for fluorescence readout is the lack of signal saturation and long-lasting intensity (36, 37).

### WB Results Correlate with Published Data from LC–MS Platform

**Fig. 3:**
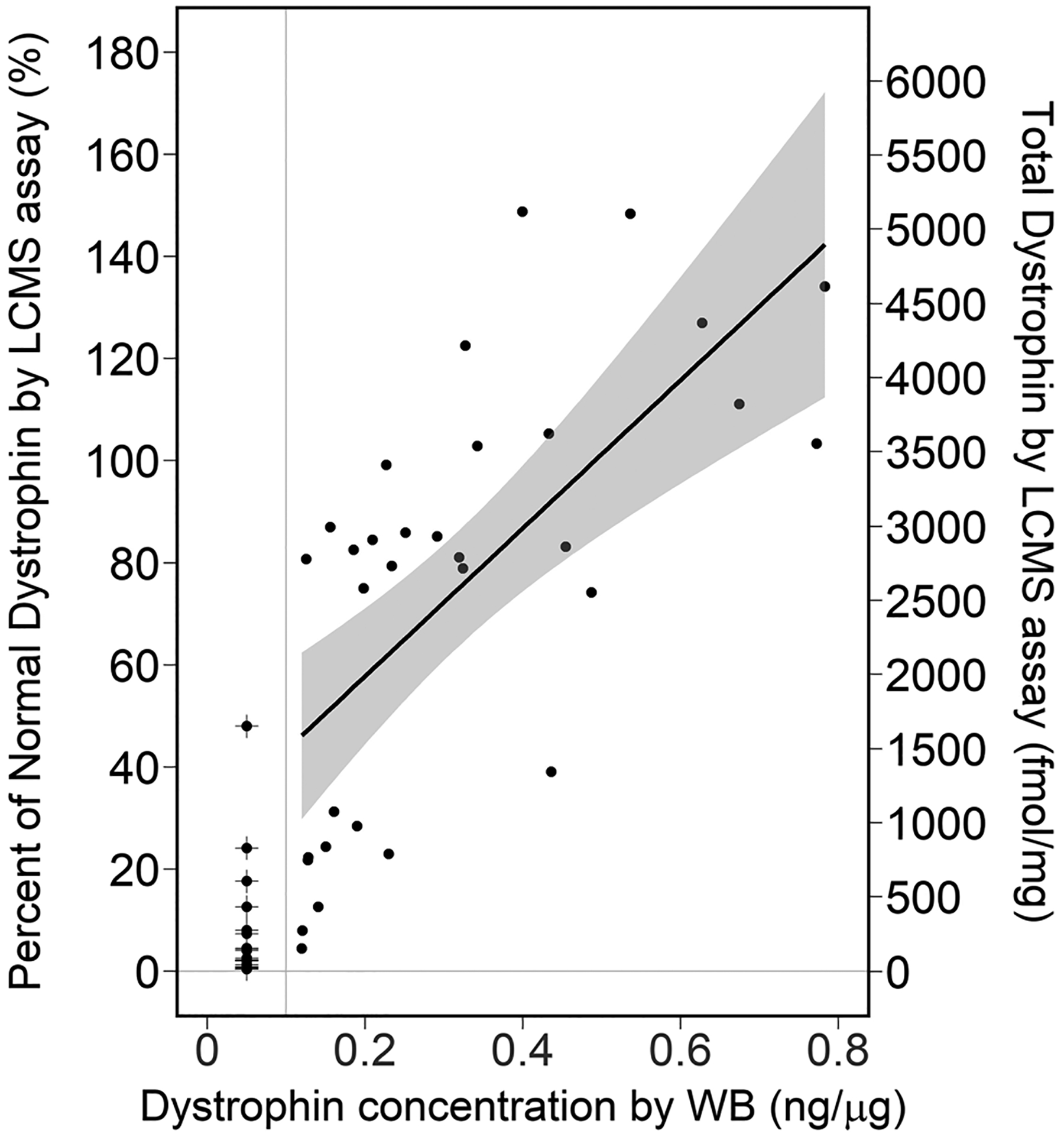
Relationship between the dystrophin concentration by Western blot and LC–MS. Linear regression for samples (*n* = 32) with quantifiable dystrophin concentrations by Western blot (WB_DYS_) and LC–MS %normal dystrophin (LC_DYS_): %normal LC_DYS_ = 28.8 + 145.1 WB_DYS_. Adjusted *R*^2^ = 0.43, *p*-value for the regression coefficient of WB_DYS_ < 0.0001. The vertical gray line is the LLOQ of 0.1 ng/μg obtained by WB. Imputed values of WB_DYS_ (*n* = 21) below the LLOQ were overlaid with +. The shaded region is the 95% confidence band

We conducted a correlation analysis on the data generated through WB and previously published LC–MS/MS measurements on the same cohort of control and dystrophinopathy samples (32). Overall, 53 samples have data from both platforms out of a total of 53 results per assay platform. Among those, 7 pairs of DMD samples were BLQ in both platforms, and 14 pairs (9 DMD, 5 BMD) of samples had BLQ results obtained by WB and quantifiable results obtained by LC–MS with a median of 4.4%. For the 32 pairs (2 DMD, 11 BMD, and 19 CTR) of samples (60%) with quantifiable results in both platforms, the Pearson and Spearman correlations were 0.67 and 0.74, respectively. Regression analyses were performed to further evaluate the relationship between the quantifiable dystrophin measures obtained by the two platforms. The adjusted *R*^2^ was 0.43 and 0.69 for the models without and with the cohort information, respectively (Fig. 3; Supplemental Fig. 2). The variability in the dystrophin levels obtained by LC–MS was partially explained by the WB dystrophin levels, and further improved by the addition of the cohort information.

It is worth noting that the published LC–MS method offers an absolute measurement of protein amount, while this WB assay is quantitative only for mini-DYS and quasi-quantitative method for the endogenous full-length DYS protein (32). In addition, the WB method uses a standard curve of mini-dystrophin as a surrogate to determine the endogenous dystrophin levels in the samples due to the size difference between DYS and mini-DYS (427 kDa *versus* 153 kDa respectively). Furthermore, the LC–MS method is one order of magnitude more sensitive compared to its WB counterpart (32).

Despite the difference between the two platforms, there was a fairly strong relationship between the results (Spearman correlation of 0.74 and fairly good model fit between WB and LC–MS for all quantifiable dystrophin levels; 100% agreement for all BLQ results and 79% of BLQ results obtained by WB were below 15% normal dystrophin obtained by LC–MS).

Overall, this data indicates that our WB method, which used mini-dystrophin as the protein standard, can serve as a reliable tool for the quantitative measurement for this truncated form of the protein while providing a quasi-quantitative measurement of endogenous full-length dystrophin in human skeletal muscle tissues within its quantitative range.

## Discussion

With the development of several potential gene therapies for treating DMD and other muscular dystrophies, there is a growing need for reliable and quantitative methods to assess dystrophin (DYS) expression in impacted muscle tissues in the clinic (44). Under these settings, DYS is used as a biomarker for assessment of therapeutic efficacy as well as a potential surrogate for muscle function (44, 45).

Here, we report the validation of a simple, sensitive, and reproducible Western blot assay that was designed to quantitate recombinant AAV mini-DYS in human skeletal muscle biopsies from clinical interventional studies. Our method utilizes a truncated form of the DYS target protein (mini-DYS) for the standard curve that matches the MW of the recombinant AAV mini-DYS protein intended for measurement in gene therapy clinical studies. The use of mini-DYS standard curve and GAPDH as internal loading controls allowed for assessment of assay precision, accuracy, and repeatability. In addition, our assay used pooled control lysates from healthy donors to circumvent the heterogeneity of DYS expression observed in DMD and BMD samples (46).

Recent advancements in detection methods, instrumentation, improvement of antibody production, and wide-scale commercialization of high-quality immunoblotting reagents (*e.g*., precast gels) have led to the transformation of Western blot technique from a labor-intensive and qualitative confirmation method to an efficient semi-quantitative tool (19, 24, 47–49).

Leveraging these technical advances, two groups recently reported the use of capillary Western immunoassay (Protein-Simple Wes) for quantitation of dystrophin protein levels in skeletal muscle tissues collected from healthy, DMD, and BMD individuals (22, 50). This automated WB approach offered several significant improvements over the traditional WB technique including reduction of manual intervention, and automation of key assay steps (protein separation, immunoblotting, and detection) to reach picogram levels of sensitivity (51). Although this technology and similar others can facilitate protein quantitation in targeted tissue lysates, they require sophisticated instrumentation that may not be easily attainable and can be cost prohibitive.

Our quantitative WB method presented here provides several improvements over other previously published immunoblotting assays used for measuring DYS in a range of dystrophinopathies (22, 50). For instance, our WB method utilizes a calibration standards of well-characterized reference material (mini-dys recombinant protein) to accurately measure the endogenous protein in contrast with previous methods which rely on ratio-metric estimation of DYS in DMD wt dystrophin samples (52, 53). In addition, QCs that cover the quantitative range of our assays are used in every analytical run to ensure that the assay performance met acceptance criteria and that only passing runs are used for data analysis. Finally, our WB method is simple, affordable, and easy to use in standard research laboratories compared to more sophisticated WB platforms (50). Therefore, this WB method offers a quantitative determination for truncated DYS (mini-DYS) and a quasi-quantitative determination for the full-length endogenous DYS in clinical testing.

In the absence of regulatory guidelines for WB assays intended for clinical use, we adhered to the best practices for validating fit-for-purpose biomarker assays (31). In total, we conducted 20 analytical runs to test assay accuracy and precision, dilution linearity, analyte stability, and dynamic range (0.5–12 ng) (Supplemental Tables I and II). Our assay was reproducible within the specified dynamic range and met the prior acceptance criteria and accuracy (%RE) ≤ 30.0% for QC samples (low, medium, high) with 42.0% RE at LLOQ of 0.5 ng, add second LLOQ, and − 4.2% at ULOQ. Furthermore, the sum of the precision (%CV) and accuracy (%RE), known as total error, was reported to be ≤ 30%, and ≤ 50% at the LLOQ2, but > 50% at LLOQ1 (Tables I, II, III, IV, and V; Supplemental Table I and II). To account for the variability of DYS in DMD and BMD tissue lysates, we spiked mini-DYS protein into lysis buffer and mixed with pooled human skeletal muscle lysate prior to heating and addition to the electrophoresis gels.

Regression and correlation analyses using patient samples from healthy, BMD, and DMD cohorts against a fully quantitative LC–MS assay and a relative quantitative WB method provide confidence that our validated Western blot assay can be used as an efficient, inexpensive, and reproducible method to measure DYS in skeletal muscle. Although this WB method does not achieve the level of sensitivity of our previously published LC–MS method, and lacks the wider dynamic range and absolute quantitation for endogenous dystrophin in muscle tissue extracts (32), it is suitable for measuring moderate levels of dystrophin (as in BMD) and moderately well-expressed levels of miniaturized dystrophin following gene therapy treatments.

In conclusion, this body of work contributes to ongoing industry efforts to establish harmonized guidelines for validation of Western blotting methods intended for clinical use and supports clinical sample testing across industry and academic institutions.

## Supplementary Material

Supplementary file 1

Supplementary file 2

Supplementary file 3

## Figures and Tables

**Table I:** Performance of Calibration Curve Parameters for Mini-Dystrophin Calibration Standards in Human Skeletal Muscle Lysate

Parameter	0.00 ng	0.102 ng	0.256 ng	0.640 ng	1.60 ng	4.00 ng	10.0 ng	15.0 ng
Mean	0.0265	0.0943	0.24	0.649	1.72	4.12	9.48	15.5
S.D	0.0355	0.0240	0.039	0.058	0.13	0.30	0.61	0.6
%CV	134	25.5	16.4	9.00	7.60	7.30	6.40	3.60
%Bias		−7.5	−6.3	1.4	7.5	3.0	−5.2	3.3
%TE		33.0	22.7	10.4	15.1	27.3	11.6	6.9
*n*	10	14	20	20	20	20	20	20

*SD*, standard deviation; *CV*, coefficient of variation; *TE*, total error

**Table II:** Analytical Performance of Mini-Dystrophin Quality Control Samples in Human Skeletal Muscle Lysate

Parameter	LLOQ (0.5 ng)	LLOQ (1.0 ng)	Low (2.5 ng)	Mid (5.0 ng)	High (8.0 ng)	ULOQ (12.0 ng)
Mean	0.71	1.08	2.60	5.05	8.40	11.5
S.D	0.16	0.25	0.54	0.70	1.12	1.57
%CV	22.4	22.8	20.8	13.9	13.3	13.7
%Theoretical	142	108	104	101	105	95.8
%Bias	42.0	8.00	4.00	1.00	5.00	− 4.20
%Total error	64.4	30.8	24.8	14.9	18.3	17.8
*n*	4	4	20	20	20	4

Overall %CV 17.8

*SD*, standard deviation; *CV*, coefficient of variation; *LLOQ*, Lower Limit of Quantitation; *ULOQ*, Upper Limit of Quantitation

**Table III:** Dilutional Linearity of Individual Human Donor of Mini-Dystrophin in Skeletal Muscle Lysate

Sample	Amt loaded to gel (ng)	Dilution factor (DF)	Calculated Amt (ng × DF)	% Recovery to neat
1 ng	1.0	1	1.48	148
	0.50	2	1.32	132
	0.25	4	1.08	108
	0.10	10	1.16	116
	0.05	20	1.40	140
16 ng spike	16.0	1	18.1*	113
	8.00	2	14.1	88.1
	4.00	4	14.6	91.3
	1.60	10	16.8	105
	0.80	20	13.3	83.1
50 ng spike	50.0	1	45.9*	91.8
	25.0	2	42.0*	84.0
	12.5	4	58.0	116
	5.00	10	68.5	137
	2.50	20	71.1	142

* Extrapolated values outside assay’s ULOQ; values are approximated given that precision and accuracy were not assessed at protein levels > 16 ng

**Table IV:** Freeze–Thaw and Bench Top Stability

		4 freeze–thaw cycles		Benchtop at 2 h		1 month		7 months	
Sample name	Nominal conc, ng/5 μg	Back-calculated	%RE	Back-calculated	%RE	Back-calculated	%RE	Back-calculated	%RE
Low1	2.5	1.98	− 20.8	2.84	13.6	2.26	− 9.6	2.00	− 20.0
Low2	2.5	1.90	− 24.0	3.15	26.0	2.34	− 6.4	1.93	− 22.8
Low3	2.5	1.98	− 20.8	3.17	26.8	2.37	− 5.2	2.33	− 6.8
High1	8.0	6.15	− 23.1	10.40	30.0	6.64	− 17.0	10.7	33.8
High 2	8.0	6.87	− 14.1	9.41	17.6	6.71	− 16.1	9.96	24.5
High 3	8.0	6.19	− 22.6	9.58	19.8	6.69	− 16.4	10.1	26.3

%RE is calculated as ((calculated value − nominal value) / nominal value) × 100%

**Table V:** Summary Statistics of the Dystrophin Concentration Quantified by Western Blot in Human Skeletal Muscle Samples

Cohort, *N=* number of subjects	Age of subjects (years)		Proportion of subjects with dystrophin below 0.1 ng/μg (BLQ)	Dystrophin concentration (ng/μg)			Relative to average of control group (%)		
	Median (min, max)	Mean±SD		Median (min, max)	Mean±SD	Bootstrap 95% confidence interval for the mean*	Median (min, max)	Mean ± SD	Bootstrap 95% confidence interval for the mean*
DMD *n* = 20	6 (3, 10)	6 ± 1.8	90%	0.05 (0.05, 0.14)	0.06 ± 0.03	(0.05, 0.07)	9.1 (8.6, 24.2)	12.6 ± 4.8	(11, 15)
BMD *n* = 18^†^	8 (3, 19)	8 ± 4.3	33%	0.13 (0.05, 0.44)	0.14 ± 0.1	(0.10, 0.19)	34.5 (13.9, 87.4)	35.8 ± 21.3	(27, 46)
CTRL *n* = 20	9 (4, 16)	10 ± 3.8	0%	0.33 (0.16, 0.78)	0.40 ± 0.19	(0.32, 0.49)	80.1 (38.8, 188.0)	91.2 ± 42.2	(74, 110)

* Random number generator state (random seed) was set at 2020 in R version 4.0.3 (55); results based on 10,000 bootstrap samples of each cohort.

† Two of the 20 BMD patient muscle biopsy extracts did not have valid results (1 sample was not recognized by DYSB; another sample was insufficient to be analyzed)

## Data Availability

The data generated during and/or analyzed during the current study are included in the main results section and supplementary materials. Any additional source data available may be provided upon reasonable request from the corresponding author and as permitted by corporate polices of the authors.

## Abbreviations

BLQ: Below the limit of quantitation
BMD: Becker muscular dystrophy
DMD: Duchenne muscular dystrophy
DYS: Dystrophin
F/T: Freeze/thaw
GAPDH: Glyceraldehyde-3-phosphate dehydrogenase
IHC: Immunohistochemistry
LC-MS: Liquid chromatography–mass spectrometry
WB: Western blot
WT: Wild type
