# Supplementary material for: Development and Validation of a Western Blot Method to Quantify Mini-Dystrophin in Human Skeletal Muscle Biopsies
Source: AAPS J. Author manuscript; Available in PMC 2023 Mar 23. (PMC10034579; doi:10.1208/s12248-022-00776-0)
Supplement: Supplementary file 1 [file NIHMS1883240-supplement-Supplementary_file_1.docx]

## Supplemental table I: Calibration Curve Parameters for Mini-Dystrophin Calibration Standards in Human Skeletal Muscle Lysate

| Curve Number | Min | Max | Slope | Ed50 | R-Squared | LLOQ | ULOQ | Regression Footnote(s) |
| --- | --- | --- | --- | --- | --- | --- | --- | --- |
| 27 | -0.00252 | 3.69 | 0.892 | 222 | 0.9947 | 0.500 | 10.0 | 1 |
| 28 | -0.00164 | 188 | 0.996 | 5780 | 0.9990 | 0.500 | 10.0 | 1 |
| 29 | -0.00557 | 650 | 1.11 | 938 | 0.9953 | 0.500 | 10.0 | 1 |
| 30 | -0.000519 | 14.7 | 1.03 | 582 | 0.9951 | 0.500 | 10.0 | 1 |
| 31 | 0.00489 | 186 | 1.22 | 977 | 0.9428 | 0.500 | 10.0 | 1 |
| 32 | 0.00523 | 3.42 | 1.28 | 39.8 | 0.9969 | 0.500 | 10.0 | 1 |
| 33 | -0.00683 | 19.7 | 0.949 | 662 | 0.9751 | 0.500 | 10.0 | 1 |
| 34 | 0.000465 | 67.7 | 0.983 | 1970 | 0.9572 | 0.500 | 10.0 | 1 |
| 35 | 0.00140 | 1.08 | 1.08 | 31.9 | 0.7692 | 0.500 | 10.0 | 1 |
| 37 | -0.00105 | 22.4 | 1.11 | 570 | 0.9926 | 0.500 | 10.0 | 1 |
| 38 | 0.00368 | 1.53 | 1.48 | 17.2 | 0.9709 | 0.500 | 10.0 | 1 |
| 47 | -0.00711 | 77.2 | 0.965 | 1420 | 0.9901 | 0.500 | 12.0 | 1 |
| 39 | 0.000307 | 267 | 1.10 | 15600 | 0.9956 | 0.500 | 10.0 | 1 |
| 49 | -0.000725 | 153 | 1.03 | 2270 | 0.9984 | 0.500 | 12.0 | 1 |
| 40 | 0.000166 | 2.25 | 0.988 | 66.4 | 0.9993 | 0.500 | 10.0 | 1 |
| 41 | 0.000263 | 12.5 | 0.996 | 406 | 0.9944 | 0.500 | 10.0 | 1 |
| 42 | 0.0000883 | 4.08 | 1.14 | 21.4 | 0.9980 | 0.500 | 10.0 | 1 |
| 43 | 0.00125 | 243 | 1.02 | 1520 | 0.9705 | 0.500 | 10.0 | 1 |
| 44 | 0.00682 | 1.74 | 1.43 | 14.3 | 0.9129 | 0.500 | 10.0 | 1 |
| 46 | 0.000460 | 9220000 | 1.12 | 20800000 | 0.9894 | 0.500 | 10.0 | 1 |
|  |  |  |  |  |  |  |  |  |
| Mean | -0.0000472 | 461000 | 1.10 | 1040000 | 0.9719 |  |  |  |
| S.D. | 0.00366 | 2060000 | 0.154 | 4650000 | 0.0528 |  |  |  |
| %CV | -7754.2 | 446.9 | 14.0 | 447.1 | 5.4 |  |  |  |
| n | 20 | 20 | 20 | 20 | 20 |  |  |  |

1) Resp. = (Min - Max) / (1 + (Conc. / Ed50) ** Slope) + Max

**Supplemental table II:** Summary of Western Blot assay validation for the Quantitation of Mini-Dystrophin in Human Skeletal Muscle Lysate

| **Method Description** | Western blot | |
| --- | --- | --- |
| **Analyte** | Mini-Dystrophin | |
| **Matrix** | Human Skeletal Muscle Lysate | |
| **Sample Storage Temperature** | -70ºC | |
| **Detection antibody** | Leica Biosciences DYSB monoclonal antibody | |
| **Diluent Solution** | DMD Lysis buffer | |
| **MRD** | N/A | |
| **Detection Method** | Fluorescence | |
| **Sample Aliquot Volume** | 10 cryo slices of 10 µm muscle biopsy | |
| **Regression, Weighting** | Logistic auto estimate, 1/y weighting | |
| **Quantification** | Licor Odyssey CLX Image Analyzer | |
| **Calibration Range** | 0.102 to 15 ng | |
| **ULOQ** | 15 ng | |
| **LLOQ** | 0.5 ng | |
| **Validation Sample Concentrations** | 0.5, 1.0, 2.5, 5.0, 8.0, 12.0 ng | |
| **Assay Performance** |  | |
| **Overall Statistics** | Precision (%CV) | Accuracy (%RE) |
|  | 13.9 to 23.2 (all runs) | -4.2 to 42.0 (all runs) |
|  | 8.1 to 22.9 (P&A runs only) | -6.8 to 42.0 (all runs) |
| **Human Matrix** |  | |
| Skeletal muscle from 20 normal donors | 0.16 to 0.78 ng/ µg, average of 0.40 ng/µg | |
| Skeletal muscle from 20 Becker's Muscular Dystrophy donors | 0.12 to 0.43 ng/µg, 6 of 19 were BLQ | |
| Skeletal muscle from 20 Duchenne's Muscular Dystrophy donors | 0.12 to 0.14 ng/µg, 18 of 20 were BLQ | |
| **Parallelism** | linear to 1:4 in 3 of 3 samples, 1:20 in 2 of 3 samples | |
| **Stability** |  | |
| **Benchtop** | 2 hours room temperature | |
| **Freeze-thaw** | 4 freeze/ thaw cycles at -70°C | |
| **Storage stability** | 7 month at -70ºC | |
