# Supplementary figures and images for: Development and Validation of a Western Blot Method to Quantify Mini-Dystrophin in Human Skeletal Muscle Biopsies
Source: AAPS J. Author manuscript; Available in PMC 2023 Mar 23. (PMC10034579; doi:10.1208/s12248-022-00776-0)

## Slide 1
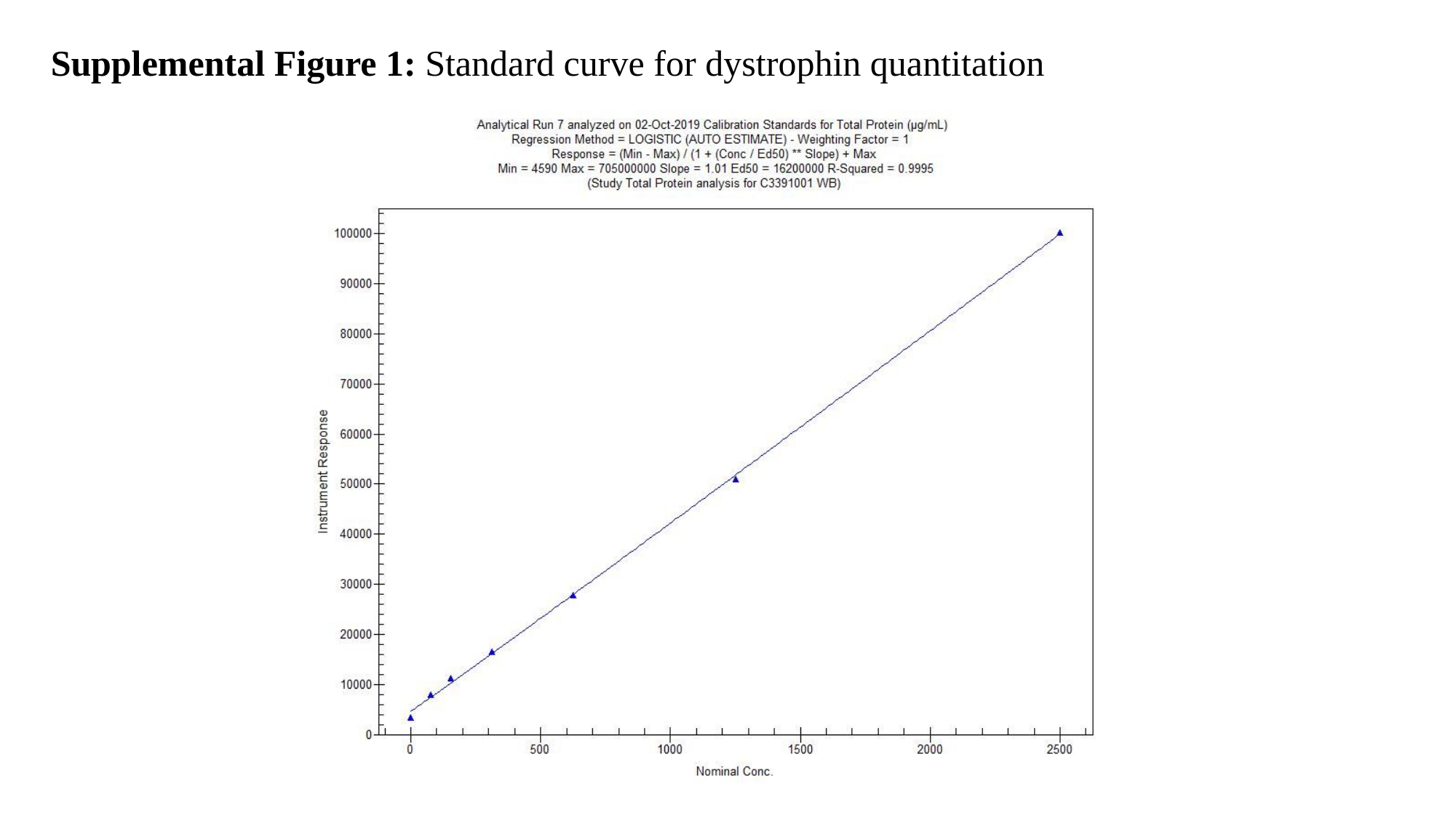

Supplemental Figure 1: Standard curve for dystrophin quantitation

Supplement: Supplementary file 2 [file NIHMS1883240-supplement-Supplementary_file_2.pptx]
