# Supplementary material for: Development and Validation of a Western Blot Method to Quantify Mini-Dystrophin in Human Skeletal Muscle Biopsies
Source: AAPS J. Author manuscript; Available in PMC 2023 Mar 23. (PMC10034579; doi:10.1208/s12248-022-00776-0)

**Supplemental Material.**

**Supplemental Figure 2. Relationship between the dystrophin concentration by Western blot and LC-MS including samples including Cohort information.**

Linear regression for all samples (n=32) with quantifiable ystrophin concentrations by Western blot (WB_DYS_) and LC-MS %normal dystrophin (LC_DYS_):

%normal LC_DYS_ = 1.9 + 64.3 × WB_DYS_ + 25.7 × BMD + 73.6 × CONTROL, where BMD = 1 for subjects in the BMD Cohort, 0 otherwise; and CONTROL = 1 for subjects in the Healthy volunteer Control cohort, 0 otherwise. Adjusted R^2^ = 0.69, p-values for the regression coefficients of WB_DYS_ and CONTROL are 0.024 and 0.0006, respectively. The vertical gray line is the LLOQ of 0.1 ng/µg obtained by WB. Imputed values of WB_DYS_ below the LLOQ were overlaid with +. The shaded regions are the 95% confidence bands.


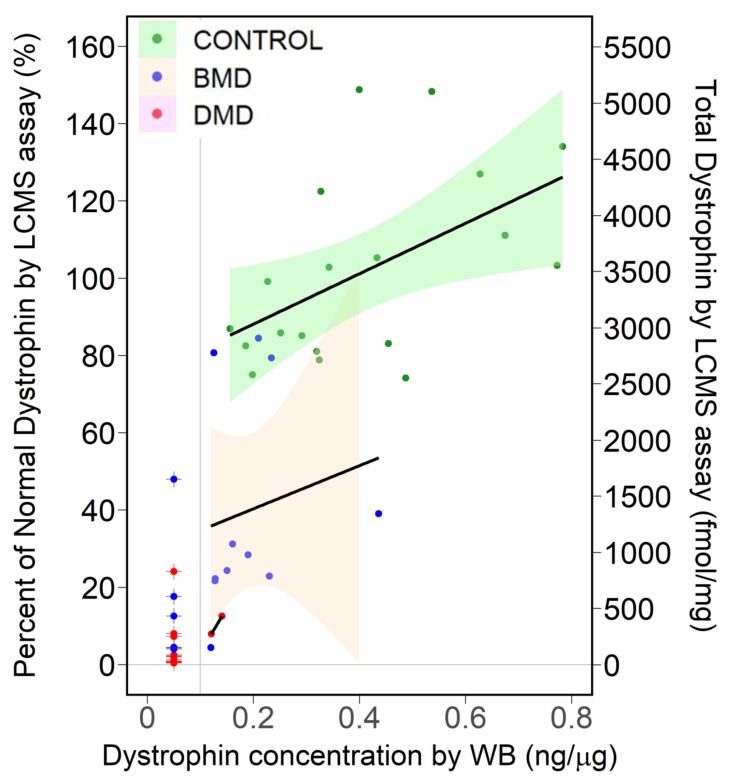

Supplement: Supplementary file 3 [file NIHMS1883240-supplement-Supplementary_file_3.docx]
